# The *Drosophila* transcriptional network is structured by microbiota

**DOI:** 10.1186/s12864-016-3307-9

**Published:** 2016-11-25

**Authors:** Adam J. Dobson, John M. Chaston, Angela E. Douglas

**Affiliations:** 1 Department of Entomology, Cornell University, Ithaca, NY USA; 2 Department of Molecular Biology and Genetics, Cornell University, Ithaca, NY USA; 3 Present address: Institute of Healthy Ageing, Department of Genetics Evolution and Environment, University College London, Gower Street, London, WC1E 6BT UK; 4 Present address: Department of Plant and Wildlife Sciences, Genetics and Biotechnology, Brigham Young University, Provo, UT 84602 USA

**Keywords:** Coexpression, *Drosophila*, Gene regulation, Microbiota, Symbiosis, Transcriptional network

## Abstract

**Background:**

Resident microorganisms (microbiota) have far-reaching effects on the biology of their animal hosts, with major consequences for the host’s health and fitness. A full understanding of microbiota-dependent gene regulation requires analysis of the overall architecture of the host transcriptome, by identifying suites of genes that are expressed synchronously. In this study, we investigated the impact of the microbiota on gene coexpression in *Drosophila*.

**Results:**

Our transcriptomic analysis, of 17 lines representative of the global genetic diversity of *Drosophila*, yielded a total of 11 transcriptional modules of co-expressed genes. For seven of these modules, the strength of the transcriptional network (defined as gene-gene coexpression) differed significantly between flies bearing a defined gut microbiota (gnotobiotic flies) and flies reared under microbiologically sterile conditions (axenic flies). Furthermore, gene coexpression was uniformly stronger in these microbiota-dependent modules than in both the microbiota-independent modules in gnotobiotic flies and all modules in axenic flies, indicating that the presence of the microbiota directs gene regulation in a subset of the transcriptome. The genes constituting the microbiota-dependent transcriptional modules include regulators of growth, metabolism and neurophysiology, previously implicated in mediating phenotypic effects of microbiota on *Drosophila* phenotype. Together these results provide the first evidence that the microbiota enhances the coexpression of specific and functionally-related genes relative to the animal’s intrinsic baseline level of coexpression.

**Conclusions:**

Our system-wide analysis demonstrates that the presence of microbiota enhances gene coexpression, thereby structuring the transcriptional network in the animal host. This finding has potentially major implications for understanding of the mechanisms by which microbiota affect host health and fitness, and the ways in which hosts and their resident microbiota coevolve.

**Electronic supplementary material:**

The online version of this article (doi:10.1186/s12864-016-3307-9) contains supplementary material, which is available to authorized users.

## Background

There is overwhelming evidence that healthy animals are persistently colonized by benign or beneficial microorganisms [1] and that microbial effects on host phenotype are conserved across animals [2], suggesting that the causal molecular interactions have ancient evolutionary origins. Altered patterns of host gene expression have been observed in transcriptomic and epigenomic analyses of hosts with different microbial complements, indicating that the microbiota influence a great diversity of host functions [3–5]. However, to establish the upstream regulators of these changes, systematic analyses of transcriptome regulation are required. Such analyses of the architecture of the host transcriptome can reflect whether the microbiota affect the overall function of the host gene-regulatory machinery, structuring the flow of information through the signaling networks that coordinate animal phenotype.

Despite the many studies of the effects of the microbiota on transcript abundance, their impact on gene coexpression (i.e., transcriptional networks) has not been investigated. Studies of transcript abundance and gene coexpression both rely on gene expression data, but address distinct questions: The object of gene coexpression analysis is not gene expression, but the coordination of the expression of suites (or “modules”) of genes [6–10]. Differential coexpression analysis (DiffCoEx) identifies modules of genes that show similar changes in coexpression (either relative to each other, relative to the rest of the transcriptome, or both) between conditions, isolating regulatory patterns that are altered or preserved. This approach recognizes that the expression of any one gene is potentially subject to reciprocal interactions from any other gene in the transcriptome, and also mechanisms that govern the expression of many genes (e.g., transcription factors, chromatin modifications, DNA methylation). Therefore, coexpression analysis reflects the transcriptome as a structure composed of circuits of co-regulated genes, accounting for potential feedbacks and pathways in expression, expanding on the view given by analyses of transcript abundance, and revealing patterns of altered transcriptome regulation. Understanding how the microbiota influences host gene-coexpression networks would therefore complement previous analyses of microbiota-dependent changes in gene expression, and provide a powerful indicator of a microbial influence on mechanisms of gene regulation. Microbial influence on the host at this gene regulatory level would be consistent with ancient origins of host-microbe interactions.

The fruit fly *Drosophila melanogaster* is ideally suited for the study of both host-microbe interactions and the architecture of transcriptional networks. The gut microbiota has been characterized, and is readily manipulated [11]. In particular, the microbiota can be eliminated (generating axenic flies) or standardized (gnotobiotic flies), and the presence and composition of the microbiota has robust effects on the fly phenotype [12, 13], with linked effects on host gene expression, especially metabolic and immune genes [11, 14]. Furthermore, *Drosophila* has superb genomic resources, including panels of genetically-variable isolines [15, 16], facilitating analysis of global responses to microbiota treatments.

The goal of this study was to determine the impact of the gut microbiota on the overall architecture of the host transcriptional network. Specifically, by defining patterns of *Drosophila* gene coexpression - rather than studying levels of expression of individual genes as in preceding studies [4, 17, 18] - we interrogated the host transcriptome for a signal that the regulatory mechanisms which coordinate transcription are microbiota-dependent. We studied the transcriptome of male *Drosophila* from genetically diverse lines originating from five continents, in order to obtain sufficient variation in gene expression to identify gene co-regulation by pairwise correlations and, thereby, to infer transcriptional networks. This genetic variation gives assurance that our results would be representative of the global diversity of *Drosophila* (and not specific to an individual genotype or strain). All but one *Drosophila* line (Canton S) were isolines, to ensure that any systematic differences between axenic and gnotobiotic flies could not be attributed to host genetic differences confounding the two microbiological treatments. To further standardize the experimental design, we compared gnotobiotic flies (i.e., with a defined microbiota) to axenic flies (i.e., lacking microbiota), so excluding the known effects of host genotype and stochastic variation on microbiota composition [19, 20].

Our analysis reveals transcriptional modules in *Drosophila*, within which network structure (measured as coexpression of groups of genes) is enhanced in the presence of the microbiota, relative to the hosts’ intrinsic baseline. In other words, symbiosis is required for high levels of gene coexpression amongst multiple *D. melanogaster* transcriptional modules. These results are consistent with a microbial influence on the host at the level of mechanisms of transcriptome regulation.

## Results

Before conducting the transcriptional network analysis of *Drosophila*, genes that were differentially expressed between axenic (germ-free) and gnotobiotic flies (with standardized microbiota) were identified. Our results (Additional file 1: Text S1; Additional file 2: Table S1; Additional file 3: Figure S1 and Additional file 4: Table S2) are fully congruent with the published studies of microbiota effects on gene expression in laboratory strains [4, 17, 18], indicating that our analysis of the architecture of the *Drosophila* transcriptome (described below) in a genetically diverse panel of wild-type *Drosophila* is relevant to the various laboratory strains used in previous studies.

To address the extent to which gene coexpression (i.e., network structure) of the *Drosophila* transcriptional network is microbiota-dependent, we devised a gene coexpression metric, the distribution of which reflects the tendency of pairs of transcripts to be co-regulated (0 = no co-regulation, 1 = perfect co-regulation), and calculated it for all gene-gene pairs in the transcriptome. The distribution of the coexpression metric differed between axenic and gnotobiotic flies (Kolmogorov-Smirnov test D = 0.0191, *p* < 2.2e-16), and median transcriptome-wide coexpression was reduced in axenic flies relative to gnotobiotic flies (Wilcoxon rank-sum test W = 3.1e16, *p* < 2.2e-16), suggesting that microbiota promotes structure in the *Drosophila* transcriptional network.

**Fig. 1:**
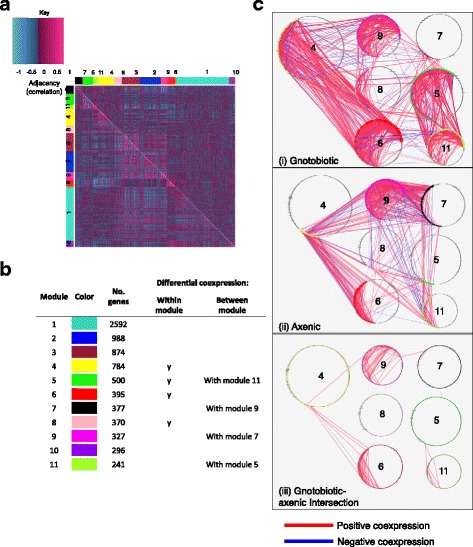
The microbiota changes transcriptional network structure in specific modules. **a** The heatmap shows the structure of pairwise gene-gene correlations amongst the *Drosophila* lines. Gene-gene coexpression is significantly greater in gnotobiotic flies than in axenic flies (Wilcoxon rank-sum, *p* < 0.00001). The largest changes in transcript-transcript correlations between axenic (*top-right*) and gnotobiotic (*bottom-left*) conditions are confined to specific transcriptional modules. Genes are organized by assignments to modules, represented by the colored side-bars. **b** Description of each module (Additional file 5: Table S3 for details). Significantly differential coexpression was determined by permutation testing (Additional file 6: Table S4). "Y" indicates differential coexpression within a module. **c** Network diagrams of differentially expressed transcriptional modules. Nodes represent genes, numbered by module assignment. Edges represent correlations between nodes above/below a threshold of Spearman's *rho* 0.75 or −0.75

We reasoned that the microbiota-dependence of gene coexpression may reflect diminished transcriptome structure in axenic flies that is either generalized across the transcriptome (i.e., linked to generalized malaise) or, alternatively, specific to certain transcriptional modules. To discriminate between these alternatives, we performed differential coexpression (DiffCoEx) analysis [21]. This analysis identifies differences in transcriptome structure between experimental conditions: genes are clustered into modules according to similarity of changes in structure (gene-gene coexpression) across conditions, and significant changes amongst these modules are isolated. Of the 16,292 genes submitted to DiffCoEx analysis, 7,744 were assigned to one of 11 modules (Fig. 1a, Additional file 5: Table S3). Permutation testing revealed that seven of these modules, containing 2,994 genes (39% of genes assigned to modules and 18% of the analyzed transcriptome), were differentially coexpressed (DC) between gnotobiotic and axenic flies (Fig. 1b, Additional file 6: Table S4). Differential network structure took two forms: four modules (4, 5, 6, 8), collectively containing 1,169 genes, showed significant changes in within-module structure upon elimination of the microbiota; and two pairs of modules (5 & 11, and 7 & 9), collectively containing 1,011 genes, showed significant changes in gene-gene correlations between modules (Additional file 6: Table S4). Consistent with transcriptome-wide patterns indicating enhanced structuring of host gene expression by the microbiota, most of these changes represented more strongly directed coexpression in gnotobiotic flies relative to axenic flies (Fig. 1c). This result cannot be explained by the microbiota suppressing variation in gene expression, because this variation in expression was not greater in axenic flies than in gnotobiotic flies (Additional file 7: Figure S2). However, coexpression of genes in module-7 and module-9 was enhanced in axenic flies relative to gnotobiotic flies (Fig. 1c), suggesting that certain host transcriptional networks are suppressed by the microbiota. Mean expression of each module is presented in (Additional file 8: Figure S3). This analysis demonstrated that elimination of the microbiota both de-structures and re-structures the fly transcriptional network.

**Fig. 2:**
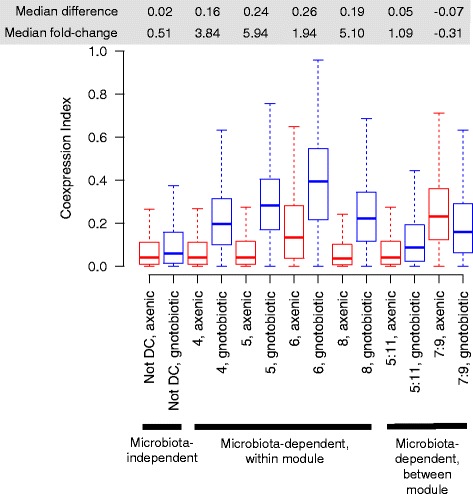
Microbiota generally increases gene coexpression above host-intrinsic capacity. The box plots show the strength of network structure (distributions of the coexpression index: unsigned squared Spearman's rho) for microbiota-independent modules (grouped together: the null distribution of coexpression, not differentially coexpressed (not DC)), each module with microbiota-dependent coexpression within the module, or between pairs of modules. For all microbiota-dependent modules except between modules 7 & 9, gene-gene coexpression is greater in gnotobiotic flies relative to axenic flies and microbiota-independent modules, indicating that the microbiota promotes network structure above the host's intrinsic capacity

Our network analysis revealed distinct microbiota-dependent and microbiota-independent transcriptional modules in axenic and gnotobiotic flies. Identifying the sign of these differences between axenic and gnotobiotic flies allowed us to ask whether the effects of microbiota on network structure represented enhanced coexpression in gnotobiotic flies, or diminished coexpression in axenic flies. These alternatives could be distinguished because microbiota-independent modules represent a null model of coexpressed modules: coexpression in these modules did not differ significantly between axenic and gnotobiotic flies and therefore is representative of the host's intrinsic baseline, independent of the influence of the microbiota. We therefore analyzed whether coexpression in DC modules was greater than the null in gnotobiotic flies (gain of coexpression), or less than the null in axenic flies (loss of coexpression). Coexpression was enhanced in DC modules relative to non-DC modules in gnotobiotic flies, but was equivalent across modules in axenic flies (Fig. 2). This result indicates that the microbiota enhances the structure of specific transcriptional subnetworks above the host's intrinsic baseline.

Having established that the microbiota enhances structure in specific transcriptional networks, we investigated the functions associated with those networks, first at the level of specific key genes. The DC modules contained many genes that are evolutionarily conserved and key regulators of growth and metabolism. The genes assigned to DC modules (Additional file 5: Table S3) code for proteins including the insulin receptor *InR,* along with its substrate *chico* and adaptor *Lnk*; jelly belly (an activator of Pi3K signaling, along with the insulin receptor); the TOR pathway members *Thor*, *Rheb* and *Rag* proteins; Spätzle processing enzyme; the lipase *Brummer*; *sugar baby*; sterol regulatory element binding protein (*SREBP*) and *SREBP cleavage activating protein*; maltases; multiple growth factors; glucose dehydrogenase; the glucose transporter *Glut1* and the downstream energy sensing kinase *AMPK*; transcription factors including the IMD factor *Relish*; the receptor of adipokinetic hormone (the invertebrate analogue of glucagon) *AkhR*; members of the RNA polymerase complex, and many more genes with known fundamental roles in coordinating cellular signaling, growth and metabolism. Taken together, these results indicate that the microbiota promotes the coexpression of many genes contributing to major signaling pathways.

**Fig. 3:**
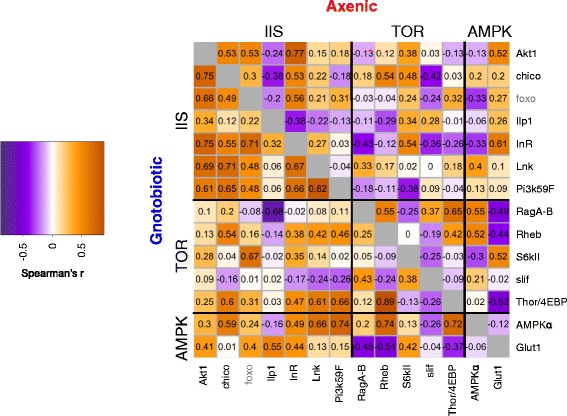
Genes in nutrient signaling networks require microbiota for structured coexpression. Modules exhibiting microbiota-dependent structure included genes with key roles in the IIS/TOR/AMPK nutrient signaling network. Pairwise coexpression (Spearman's Rho) of genes in the IIS/TOR/AMPK network [38, 39] is presented, for genes in differentially coexpressed modules. FoxO was not clustered into a DC module, but is plotted in the figure since it is the major transcriptional output of IIS. Numbers in each cell represent correlation coefficients. The figure demonstrates that the microbiota affects the regulation of these networks at the level of structured gene coexpression

To investigate the effect of the microbiota on gene coexpression further, we focused on the insulin-like signaling (IIS) and target of rapamycin (TOR) signaling pathways, which together play a key role in coordinating the response of larval growth and adult physiology to nutrient availability. Genetic lesions in these pathways have previously been shown to alter the physiological response of *Drosophila* to elimination of the microbiota [22, 23]. We found six components of IIS and five components of TOR amongst the differentially coexpressed modules. IIS in *Drosophila* consists of circulating insulin-like peptides (ILPs) which are sensed by the insulin receptor *InR*, resulting in phosphorylation and nuclear exclusion of the transcription factor dFOXO. IIS components amongst DC modules included *Ilp1*, *InR* and intracellular proteins, but not *FoxO*, suggesting that the interactive effects of microbiota and IIS on *Drosophila* phenotype are mediated at the level of disrupted coexpression of *InR* and components of the downstream Pi3K pathway (Fig. 3). The components of TOR which were amongst the differentially coexpressed modules were *Slif*, *RagA-B*, *Rheb*, and *Thor* (Fig. 3)*.* Since amino acid sensing by *Slif* feeds into TOR to regulate multiple outputs including the elongation factor *Thor*, this result suggests a requirement for the microbiota to coordinate translation with amino acid availability. Corresponding to altered coexpression of components within each pathway, more genes were negatively coexpressed between IIS and TOR in axenic flies than in gnotobiotic flies. This may be due to changes to the availability of dietary protein relative to carbohydrate in axenic flies [20, 24]. Altogether our results demonstrate that the microbiota regulate the coexpression of genes in these pathways.

To gain an overview of the possible functional impact of the microbiota's role in network structuring, we tested for enrichment of GO terms associated with each module (Additional file 9: Table S5). This analysis indicated distinct functional roles for each of the differentially coexpressed modules, and that specific DC modules regulate specific processes in a microbiota-dependent manner. These processes included module-specific regulation of mitosis (module 7), lipid metabolism (module 5), metabolism (modules 11, 8 and 9), neurophysiology and behaviour (module 6), and gene expression and nucleic acid metabolism (module 4). The only GO term under-represented in any individual module relative to the genomic background was translation, with respect to module 5. Collectively, the microbiota-dependent modules were under-represented in genes involved in mRNA binding. The strong over-representation of GO terms in specific DC modules, and the parallel deficit of under-representation, revealed no evidence for functions that are specifically insulated from the effect of the microbiota, and that biological changes associated with perturbation of the microbiota can be associated specifically with changes to gene coexpression in distinct transcriptional modules. Extensive further study is needed to fully evaluate how the coexpression of genes in these pathways manifests at the level of host phenotypes. Nevertheless, our results show that the coordination of regulatory networks with evolutionarily conserved roles in matching growth to nutrient availability are promoted by the microbiota, relative to the condition in axenic flies and in transcriptional modules that are independent of the microbiota.

## Discussion

Axenic animals provide a powerful tool to study how resident microorganisms influence the phenotype of their animal hosts [11, 25]. In this study, we identified effects of microbiota on gene coexpression in 18% of the host transcriptional network, revealing that the flow of information in *Drosophila* transcriptional networks is microbiota-dependent.

A key aspect of the structure of the *Drosophila* transcriptome revealed in this study is that, in microbiota-dependent modules, network structure (i.e., gene coexpression) is enhanced in gnotobiotic flies, relative to both microbiota-independent modules in gnotobiotic flies, and the entire transcriptional network in axenic flies. In other words, the microbiota enhances the coordination of gene expression within and among particular transcriptional modules, promoting gene coexpression above levels observed in the absence of the microbiota. This observation may be indicative of more directed function in gnotobiotic flies of the mechanisms coordinating transcription of multiple genes. The phenotypic consequences of such a change to the architecture of the transcriptome remain to be established. This enhanced coexpression may be due to an adaptive benefit to one or both of the host and microbiota; or a relaxation of a constraint on the host in the presence of the microbiota, permitting enhanced coexpression amongst specific modules.

The microbial influence on the overall architecture of the transcriptome is specific to particular modules of coexpressed genes. Of the 11 modules identified, four modules (comprising 61% of genes in the network) showed no significant structural alterations between gnotobiotic and axenic flies, but the seven modules that did exhibit microbiota-dependent structure are dominated by genes with metabolic and immunological functions. These results are congruent with published evidence for microbial effects on vitamin, sugar and amino acid nutrition [13, 24, 26–28], as well as immune responses, digestion and gut cell division [13, 17, 29–31]. Altered coexpression of metabolic and signaling regulators may account for data showing that the effects of dietary change on nutrient stores are exaggerated in axenic flies, relative to flies with an unmanipulated microbiota [13]. The apparent module-specific influence of the microbiota, and the observation that coexpression is elevated in gnotobiotic flies, suggests that the activity of specific transcriptional regulators is enhanced by the microbiota. In turn, this suggestion implies that animals have evolved adaptive responses to specific signals, resulting in the observed effects of the microbiota on animal phenotype.

To our knowledge, this study is the first to show that that the microbiota influences the transcriptional network of an animal, since previous work has focused on the expression level of individual genes. These results indicate that the microbiota can direct the function of yet-unidentified regulators of gene expression. To identify causal gene-regulatory mechanisms requires identification of upstream endocrine and metabolic cues, including transcriptome-regulatory infochemicals. Furthermore, the magnitude and direction of these effects may vary with sex and diet (this study focused on male flies raised on a relatively rich diet) and between different organs of the body (this study was conducted on whole bodies).

Our findings raise two key questions of general significance: Why does the microbiota influence structure of only a subset of transcriptional modules; and why should these changes tend towards enhancing transcriptional coexpression? We hypothesize that the microbiota-dependence of coexpression in specific transcriptional modules is the result of host-microbiota coevolution, potentially involving microbial manipulation of host transcription, host reinforcement of transcription to control against microbial manipulation, and mutually beneficial transcriptional responses to symbiosis [32]. Further studies to compare the costs and adaptive benefits of variation in the strength of gene coexpression are required in order to test this hypothesis. Furthermore, our results suggest that the microbiota-dependent modules play roles in regulating the evolutionarily conserved phenomena of microbiota-dependent changes in metabolic rate, growth rate and nutrient allocation [22, 23, 27, 28, 33, 34]. We propose that a greater consideration of the microbial influence on the global architecture of the transcriptome will prove to be of broad biological relevance, by enhancing our understanding of the mechanisms by which microbiota influence animal health and symbioses evolve.

## Conclusions

Our transcriptome analysis reveals that the gut microbiota of *Drosophila* influences the strength of gene coexpression for a portion of the host transcriptome. The microbiota-dependent transcriptional modules include genes known to regulate host traits in a fashion that is influenced by the microbiota, suggesting that the microbiota may influence host phenotype, not only by altering the expression of individual genes, but also by their effects on the patterns of host gene coexpression. This specificity of the effect of the microbiota on host transcriptional networks may result from animal-microbial coevolution.

## Methods

### The flies and bacteria

*Drosophila melanogaster* lines (Additional file 10: Table S6) were reared on a yeast-glucose diet (10% yeast (MP Biomedicals 903312), 10% glucose (Sigma 158968), 1.2% agar (Apex 66–103), 0.04% phosphoric acid (Sigma), and 0.42% propionic acid (Sigma)) at 25 °C on a 12:12 light cycle. Experimental cultures of all lines were prepared, reared and sampled as a single experimental block. To produce gnotobiotic and axenic flies, eggs were collected from grape juice agar plates (10% yeast, 10% glucose, 11.3% Welch’s concentrated grape juice) within 20 h of laying, washed for 5min in 0.6% sodium hypochlorite, rinsed with sterile water, and transferred aseptically with a sterilized paint brush in a laminar flow hood to 7.5 ml sterile yeast-glucose diet (also 10% yeast, 10% glucose) in 50 ml Falcon tubes at a density of ~30 eggs per vial, as in [12]. For each line, ten vials were prepared. Five vials were left without further manipulation to develop under bacteria-free conditions. In the remaining five vials, gnotobiotic flies were created by immediately inoculating the transferred eggs with 50 μl 10^8^ colony forming units (CFU) ml^−1^ of bacteria, comprising 5 species in equal proportions: *Acetobacter pomorum* DmCS_004*,* A. *tropicalis* DmCS_006, *Lactobacillus brevis* DmCS_003*, L. fructivorans* DmCS_002 and *L. plantarum* DmCS_001 [12]. Cultures were grown from glycerol stocks on modified MRS medium (reagents from Sigma unless specified otherwise): 0.2% triammonium citrate, 0.02% magnesium sulfate heptahydrate, 0.005% manganese sulfate tetrahydrate, 0.5% sodium acetate, 0.2% dipotassium hydrogen phosphate, 2% glucose, 1.25% vegetable peptone (Becton Dickinson), 0.75% yeast extract [12]) containing 1.2% agar (Apex), either aerobically (*Acetobacter* spp.) or under CO2 (*Lactobacillus* spp.). To generate the bacterial inoculum, monocultures were grown overnight at 30 °C in 10 ml modified MRS medium, with or without shaking for *Acetobacter* and *Lactobacillus* species, respectively. After one wash in mMRS, the density of each bacterial culture was determined by OD_600_, normalized to 10^8^ CFU ml^−1^, as in [12], and the five cultures were combined in equal proportion. Flies were reared and maintained in the same vials until sampling as adults at 7–9 days after eclosion.

### RNA preparation and sequencing

On dry ice (−80 °C), replicate vials were pooled and male flies sorted into chilled sterile microcentrifuge tubes containing 110 μl of 1.4 mm lysing matrix (MP Biomedicals), 500 μl buffer RLT (Qiagen), and 5 μl β-mercaptoethanol. Flies were immediately homogenized at 4.0 m/s for 30 s on a Fastprep-24 (MP Biomedicals) and frozen at −80 °C. RNA was isolated using the RNeasy mini kit (Qiagen) and prepared for sequencing with the TruSeq RNA Sample Prep Kit v2 – Set A (Illumina RS-122-2001, Additional file 11: Table S7). The samples were sequenced (50 bp single end reads) on the Illumina HiSeq 2500 platform in multiplexed pools of 6 samples per lane.

### Data analysis

Libraries were inspected for quality using FastQC (0.11.3) and aligned to *Drosophila* genome dm6 with TopHat2 (2.0.14). Between 77.6 and 92.3% of reads per library were successfully aligned. BAM files were converted to SAM files using SAMTOOLS (1.2), and reads were enumerated with HTSeq (0.6.1). A custom GTF file was used, combining regions containing multiple genes into pseudo-features. All commands used are included in Supplementary Materials (Additional file 12). This procedure aligned reads to 17,148 genes (Additional file 13: Dataset S1).

All analysis after alignment was performed in R v3.1.2. The full R script used for analysis is provided in Supplementary Materials (Additional file 12). The effect of microbiota on gene expression was modeled with DESeq, accounting for host genotype and sequencing lane as covariates. All other analyses were performed after applying the variance-stabilizing transformation (VST) included in the DESeq package [35] (Additional file 14: Dataset S2), to achieve heteroscedasticity in the reads.

Microbiota-dependent changes in the transcriptional network were analyzed by Differential Coexpression Analysis (DiffCoEx) [21], which modifies procedures from Weighted Gene Coexpression Network Analysis (WGCNA) [36]. After filtering for quality control, 12,754 genes were submitted to DiffCoEx. Briefly, adjacency matrices (signed squared Spearman's rank correlation matrices) were calculated separately for transcripts in axenic and gnotobiotic flies, and the differences between these matrices calculated. A Topological Overlap Matrix (TOM) was calculated from the matrix of correlation change. To find modules of genes exhibiting common structural changes between the two microbial conditions, genes were clustered by average hierarchical clustering, using the TOM as a distance metric. Modules were defined by the “hybrid” method of dynamic tree cutting [37], cutting the tree at a height of 0.79 (71.2% of total height range in the tree), requiring a minimum cluster size of 100 genes. Representative “Eigengene” expression values [36], determined as the first principal component of expression values of genes within each module, were used to find modules showing similar structural changes, by hierarchical clustering using Euclidian distance, and modules with Eigengenes that branched at a height of ≤0.2 were merged. Significance of within-module and between-module changes in correlation were calculated by 1000 iterations of permutation tests as in [21]. Coexpression metrics were calculated as squared Spearman's rank correlation matrices, because squared correlation matrices are the basis of our transcriptional networking approach (above). These values were not re-signed (so that the mean was not zero), such that a higher value corresponds to stronger gene-gene coexpression, irrespective of the sign of the correlation. Gene Ontology (GO) enrichment was determined by GORILLA (http://cbl-gorilla.cs.technion.ac.il/) and/or the BiNGO plugin in Cytoscape for transcriptional networks.

## Additional files

Additional file 1: Text S1. Supplementary Text: Analysis of Differential Gene Expression Patterns. (DOCX 17 kb)
Additional file 2: Table S1. Differential gene expression between axenic and gnotobiotic flies. Microbiota-dependent changes in average expression were analyzed using DESeq, with a model that included sequencing lane and fly line as cofactors. The table shows gene name in FlyBase format, fold change expression (change in fitted coefficients, log_2_ scale) and *p*-values (FDR). (DOCX 22 kb)
Additional file 3: Figure S1. Tissue tropism of differential gene expression in axenic and gnotobiotic flies. For the set of 177 genes that were differentially expressed between axenic and gnotobiotic flies, the bars show the frequency of genes which were enriched in each given tissue relative to the whole body in the FlyAtlas dataset. (PDF 103 kb)
Additional file 4: Table S2. Gene ontology enrichment, full sets of terms and differentially expressed genes for (a) biological process enriched in axenic flies, (b) molecular function enriched in axenic flies, (c) cellular component enriched in axenic flies, (d) biological process depleted in axenic flies, (e) molecular function depleted in axenic flies, (f) cellular component depleted in axenic flies. (CSV 55 kb)
Additional file 5: Table S3. Gene membership of transcriptional modules. The table shows gene identifiers and names in FlyBase format, the module to which the gene was assigned, and whether the module exhibited significantly altered network structure (differential coexpression, i.e., TRUE) between axenic flies and gnotobiotic flies. Significant differential coexpression can indicate changes within a module or between two modules, see Fig. 1. (CSV 457 kb)
Additional file 6: Table S4. Frequency of data permutations with dispersion statistic greater than observed, within individual modules and between pairs of modules. Permutation tests suggesting significant changes (≤50/1000 permutations﻿ i.e., *p* ≤0.05) are underscored. (DOCX 15 kb)
Additional file 7: Figure S2. Coefficients of variation per gene in axenic and gnotobiotic flies. Coefficients of variation across all lines were calculated per gene. The grey line indicates the null of equivalence between the two conditions. (PDF 875 kb)
Additional file 8: Figure S3. Mean expression per transcriptional module in axenic and gnotobiotic conditions. Each bar shows mean expression of all genes assigned to each given transcriptional module. Color coding corresponds to Fig. 1b. (PDF 177 kb)
Additional file 9: Table S5. GO enrichment amongst microbiota-dependent transcriptional modules. Enrichment of GO terms in microbiota-dependent modules was analyzed in Cytoscape using BINGO. The table shows transcriptional module identifiers, description of GO terms enriched (FDR ≤ 0.05) in each module, the GO term identifiers, and *p*-values (FDR). (CSV 31 kb)
Additional file 10: Table S6.
*Drosophila* lines used in this study. (DOCX 13 kb)
Additional file 11: Table S7. Adapter sequences used for RNA sequencing. (DOCX 12 kb)
Additional file 12: Data analysis tools. The unzipped folder contains (1) parameters used to align and count reads (align_count_index_reads.txt) and (2) an R script to replicate the data analysis. See README file and R script within folder for instructions. (ZIP 15304 kb)
Additional file 13: Dataset S1. Read counts per gene. Each column denotes a different sample. See Table S4 for line name information. Samples suffixed by “a” are axenic, samples suffixed by “g” are gnotobiotic. Reads aligning to genomic regions coding for multiple genes are tabulated to a feature representing the multiple possible genes. (CSV 2506 kb)
Additional file 14: Dataset S2. Variance-stabilized read counts. Dataset S1 with a variance-stabilising transformation applied using functions provided by the DESeq library. (CSV 7395 kb)

## Abbreviations

CFU: Colony-forming units
DC: Differentially coexpressed
GO: Gene ontology
SREBP: Sterol regulatory element binding protein
VST: Variance-stabilising transformation
WGCNA: Weighted Gene Coexpression Network Analysis
