# Erratum to: The Drosophila transcriptional network is structured by microbiota

**DOI:** 10.1186/s12864-017-3508-x

**Published:** 2017-02-01

**Authors:** Adam J. Dobson, John M. Chaston, Angela E. Douglas

**Affiliations:** 1 Department of Entomology, Cornell University, Ithaca, NY USA; 2 Department of Molecular Biology and Genetics, Cornell University, Ithaca, NY USA; 3 Present address: Institute of Healthy Ageing, Department of Genetics Evolution and Environment, University College London, Gower Street, London, WC1E 6BT UK; 4 Present address: Department of Plant and Wildlife Sciences, Genetics and Biotechnology, Brigham Young University, Provo, UT 84602 USA

## Erratum

In this version of this article that was originally published [1] there were errors with figure 1a with certain labels missing and other labels being misaligned.

The original article was corrected.

The publisher apologises for these errors.
