# Supplementary material for: The Drosophila transcriptional network is structured by microbiota
Source: BMC Genomics. 2016 Nov 25;17:975. doi: 10.1186/s12864-016-3307-9 (PMC5124311; doi:10.1186/s12864-016-3307-9)
Supplement: Additional file 1: Text S1. — Supplementary Text: Analysis of Differential Gene Expression Patterns. (DOCX 17 kb) [file 12864_2016_3307_MOESM1_ESM.docx]

**Supplementary Text: Analysis of Gene Expression Patterns**

The first analysis of gene expression patterns in 17 *Drosophila* lines of diverse geographic origin identified genes that were differentially expressed (DE) between axenic (germ-free) and gnotobiotic flies (with standardized microbiota). In total, 177 genes were DE (FDR ≤ 1%) between the gnotobiotic and axenic flies (Additional File 2, table S1), comprising 51 genes upregulated and 126 genes downregulated in axenic flies, relative to gnotobiotic flies. These genes had functions congruent with results from previous studies [1, 2], including various metabolic enzymes (e.g. maltases, lipases, amylases) and metabolic and immune signaling molecules, as well as antimicrobial peptides (e.g. *Defensin, Diptericin*). By reference to the FlyAtlas dataset [3], the majority of microbiota-responsive transcripts were likely enriched in the midgut (Additional File 3: Fig. S1). To gain a high-level overview of likely functional effects of these differences in gene expression, Gene Ontology (GO) enrichment analysis was performed. This analysis pointed to upregulation of lipid and sterol transport, and downregulation of immunity and nucleic acid metabolism in axenic flies (Additional File 4: Table S2). Together, the differential expression and GO analyses confirmed the effect of microbiota on mean expression of individual genes with metabolic functions, and suggested that these changes are conserved amongst genetically diverse lines.

Although the changes identified in this study between axenic and gnotobiotic flies are fully consistent with expectations based on the published literature, this study revealed relatively few genes that were differentially expressed (i.e. altered mean expression) between axenic and gnotobiotic flies across the 17 *Drosophila* lines, compared to published studies that focus on single *Drosophila* lines [1, 2, 4]. The large sample size in the present study precludes low statistical power as an explanation for this discrepancy. One possible explanation is that host genotype and the microbiota have interactive effects on host gene expression, consistent with known patterns in *Drosophila* nutritional phenotypes [5, 6]. Our study was designed to isolate only global changes in expression across the 17 genetically-diverse *Drosophila* lines, and therefore does not detect context or background-specific effects that may underlie genotype-by-microbiota statistical effects. Additionally or alternatively, some effects of the microbiota on gene expression may be tissue-specific, and changes identified in previous tissue-specific studies, particularly the gut, are potentially undetectable in the whole-fly analyses conducted here. An interesting issue for future research is the contribution of expression patterns in specific tissues – particularly the gut - to the overall architecture of the microbiota-dependent transcriptome, and the extent to which microbial metabolites and other infochemicals might contribute to long-distance effects of the microbiota on host gene expression patterns.

1. Alenghat T, Artis D: **Epigenomic regulation of host-microbiota interactions**. *Trends Immunol* 2014, **35**(11):518-525.

2. Erkosar B, Defaye A, Bozonnet N, Puthier D, Royet J, Leulier F: **Drosophila microbiota modulates host metabolic gene expression via IMD/NF-kappaB signaling**. *PLoS One* 2014, **9**(4):e94729.

3. Chintapalli VR, Wang J, Dow JA: **Using FlyAtlas to identify better Drosophila melanogaster models of human disease**. *Nat Genet* 2007, **39**(6):715-720.

4. Guo L, Karpac J, Tran SL, Jasper H: **PGRP-SC2 promotes gut immune homeostasis to limit commensal dysbiosis and extend lifespan**. *Cell* 2014, **156**(1-2):109-122.

5. Chaston JM, Dobson AJ, Newell PD, Douglas AE: **Host genetic control of the microbiota mediates Drosophila nutritional phenotype**. *Appl Environ Microbiol* 2015.

6. Dobson AJ, Chaston JM, Newell PD, Donahue L, Hermann SL, Sannino DR, Westmiller S, Wong AC, Clark AG, Lazzaro BP *et al*: **Host genetic determinants of microbiota-dependent nutrition revealed by genome-wide analysis of Drosophila melanogaster**. *Nat Commun* 2015, **6**:6312.
