# Supplementary material for: The Drosophila transcriptional network is structured by microbiota
Source: BMC Genomics. 2016 Nov 25;17:975. doi: 10.1186/s12864-016-3307-9 (PMC5124311; doi:10.1186/s12864-016-3307-9)
Supplement: Additional file 2: Table S1. — Differential gene expression between axenic and gnotobiotic flies. Microbiota-dependent changes in average expression were analyzed using DESeq, with a model that included sequencing lane and fly line as cofactors. The table shows gene name in FlyBase format, fold change expression (change in fitted coefficients, log2 scale) and p-values (FDR). (DOCX 22 kb) [file 12864_2016_3307_MOESM2_ESM.docx]

| Table S1. Differential gene expression between axenic and gnotobiotic flies | | |
| --- | --- | --- |
| Gene* | Log_2_ fold-change (Gnotobiotic / axenic)** | FDR*** |
| Vm34Ca | 4.00 | 0.00017614 |
| AttD | 3.12 | 3.74E-10 |
| edin | 3.07 | 1.75E-08 |
| Vm26Aa | 2.97 | 0.001304302 |
| CG14499 | 2.55 | <2.2E-14 |
| CG9759 | 2.36 | <2.2E-14 |
| Dro | 2.25 | <2.2E-14 |
| CG43659 | 2.21 | <2.2E-14 |
| Dpt | 2.15 | <2.2E-14 |
| CecC | 2.12 | 9.25E-06 |
| CR45045 | 2.10 | 2.26E-09 |
| CG7017 | 1.82 | 1.03E-10 |
| AttC | 1.75 | <2.2E-14 |
| AttA | 1.68 | <2.2E-14 |
| DptB | 1.60 | <2.2E-14 |
| CR44404 | 1.50 | 0.008232152 |
| pirk | 1.45 | <2.2E-14 |
| Mtk | 1.44 | <2.2E-14 |
| Def | 1.41 | 8.81E-12 |
| CG43920 | 1.29 | 0.002682053 |
| CG43348 | 1.28 | <2.2E-14 |
| CG13749 | 1.22 | 2.47E-05 |
| AttB | 1.22 | <2.2E-14 |
| GstD8 | 1.14 | 6.10E-13 |
| CG14957 | 1.13 | 0.000117237 |
| CG13947 | 1.05 | <2.2E-14 |
| CG18179 | 1.02 | 2.04E-11 |
| CG16775 | 1.01 | 1.09E-09 |
| Listericin | 1.00 | <2.2E-14 |
| CG4500 | 1.00 | 3.96E-06 |
| Fst | 1.00 | <2.2E-14 |
| lectin-37Da | 0.99 | 2.77E-10 |
| CG6967 | 0.98 | 0.001197602 |
| ade3 | 0.93 | <2.2E-14 |
| CG10912 | 0.92 | <2.2E-14 |
| Lip3 | 0.90 | 3.01E-09 |
| Cry | 0.90 | 1.37E-08 |
| Lsp1alpha | 0.86 | 0.000227612 |
| CG32335 | 0.85 | 1.31E-05 |
| CG34040 | 0.84 | 2.29E-11 |
| CG4586 | 0.84 | 0.003449492 |
| CG6271 | 0.82 | 3.43E-07 |
| CG11089 | 0.82 | 9.29E-11 |
| MtnD | 0.81 | 1.28E-08 |
| MtnE | 0.80 | 5.65E-09 |
| CG13482 | 0.78 | 1.11E-08 |
| Pxt | 0.78 | 0.004047306 |
| AdSL | 0.77 | 1.11E-08 |
| CG42751 | 0.76 | 0.00211087 |
| CG4757 | 0.76 | 9.53E-09 |
| osk | 0.74 | 0.008254213 |
| CG3999 | 0.74 | 8.51E-08 |
| Yp1 | 0.74 | 0.003489416 |
| Nmdmc | 0.74 | 2.14E-07 |
| CG42821 | 0.73 | 1.28E-05 |
| pug | 0.70 | 2.71E-08 |
| CG10824 | 0.70 | 1.16E-07 |
| CG14500 | 0.70 | 2.86E-05 |
| CG42825 | 0.68 | 6.42E-06 |
| Drs | 0.67 | 3.65E-07 |
| CG13177 | 0.67 | 3.49E-05 |
| Obp56h | 0.67 | 0.004969742 |
| CG6767 | 0.67 | 2.56E-07 |
| CG6910 | 0.66 | 3.57E-07 |
| CG34198 | 0.66 | 8.13E-05 |
| PGRP-LB | 0.65 | 2.60E-06 |
| PGRP-SC2 | 0.65 | 6.76E-06 |
| Sardh | 0.65 | 3.90E-05 |
| CG3604 | 0.65 | 1.44E-05 |
| CG9119 | 0.64 | 7.82E-07 |
| CG6277 | 0.64 | 1.51E-06 |
| CG43349 | 0.63 | 8.41E-06 |
| Mal-B2 | 0.62 | 2.52E-06 |
| CG12224 | 0.62 | 2.27E-05 |
| CG34227 | 0.62 | 1.53E-05 |
| CG43055 | 0.61 | 2.86E-05 |
| CG8679 | 0.61 | 0.001480092 |
| CG13324 | 0.61 | 8.81E-05 |
| Amyrel | 0.60 | 4.37E-05 |
| ade2 | 0.59 | 2.86E-05 |
| Ahcy13 | 0.58 | 3.48E-05 |
| CG14245 | 0.57 | 2.27E-05 |
| CG12766 | 0.57 | 0.000141994 |
| CG1246 | 0.57 | 0.000188325 |
| PGRP-SD | 0.56 | 0.000591446 |
| CG15263 | 0.56 | 0.000258378 |
| CG15043 | 0.55 | 0.000124935 |
| Drat | 0.55 | 8.81E-05 |
| bmm | 0.54 | 0.000125983 |
| Mal-A4 | 0.53 | 0.000192449 |
| Arc1 | 0.52 | 0.000191437 |
| CG7953 | 0.52 | 0.000270787 |
| CG18547 | 0.52 | 0.000316107 |
| Adgf-D | 0.51 | 0.000603273 |
| CG16965 | 0.51 | 0.001710971 |
| CG15068 | 0.51 | 0.000354308 |
| CG18748 | 0.50 | 0.005793283 |
| CG3011 | 0.50 | 0.000603273 |
| CG10910 | 0.50 | 0.001018922 |
| CG1208 | 0.49 | 0.000925022 |
| MtnA | 0.49 | 0.000587529 |
| CG3609 | 0.48 | 0.00076398 |
| Obp49a | 0.48 | 0.003935819 |
| CG11899 | 0.48 | 0.001287209 |
| CG1139 | 0.48 | 0.003970127 |
| CG7296 | 0.48 | 0.006512264 |
| CG32054 | 0.47 | 0.008254213 |
| CG13323 | 0.47 | 0.001855213 |
| lectin-28C | 0.46 | 0.003072949 |
| CG6484 | 0.46 | 0.002009113 |
| trbl | 0.46 | 0.00521941 |
| CG4607 | 0.46 | 0.002943525 |
| CG3699 | 0.45 | 0.002682053 |
| mal | 0.45 | 0.009492565 |
| Prat2 | 0.45 | 0.002896215 |
| CG10383 | 0.45 | 0.004136255 |
| Gnmt | 0.45 | 0.002759268 |
| CG6283 | 0.45 | 0.003456054 |
| CG16986 | 0.45 | 0.005033971 |
| FBgn0014903+FBgn0053082 | 0.44 | 0.005810715 |
| CG1943 | 0.44 | 0.006770686 |
| CG31148 | 0.44 | 0.008225263 |
| CG31300 | 0.44 | 0.006770686 |
| ade5 | 0.43 | 0.00555146 |
| CG8317 | 0.43 | 0.008097669 |
| tobi | 0.42 | 0.006925478 |
| Sfp24Bb | -0.44 | 0.004282112 |
| Npc2h | -0.44 | 0.006176352 |
| CG9090 | -0.44 | 0.003790959 |
| Npc2g | -0.45 | 0.003612784 |
| Lsd-1 | -0.45 | 0.003935819 |
| CG12990 | -0.47 | 0.002611816 |
| Acp76A | -0.47 | 0.002102469 |
| CG5991 | -0.49 | 0.001031321 |
| CG10592 | -0.51 | 0.001245965 |
| CG3348 | -0.51 | 0.008433919 |
| Rfabg | -0.52 | 0.000258767 |
| CG11911 | -0.52 | 0.000995845 |
| sug | -0.52 | 0.001425377 |
| Act88F | -0.53 | 0.000580026 |
| Nplp3 | -0.54 | 0.000229792 |
| CG11892 | -0.54 | 0.000124036 |
| Jabba | -0.54 | 0.000221751 |
| Jon25Biii | -0.56 | 6.89E-05 |
| Jon65Aii | -0.56 | 0.008502399 |
| CG13607 | -0.56 | 8.13E-05 |
| Sfp33A4 | -0.57 | 0.002655474 |
| Spn28F | -0.58 | 2.86E-05 |
| CG32483 | -0.59 | 0.001197602 |
| CG5150 | -0.59 | 6.04E-05 |
| CG10514 | -0.60 | 2.10E-05 |
| CG18327 | -0.60 | 0.002410442 |
| Npc2d | -0.61 | 0.00088044 |
| mag | -0.61 | 8.41E-06 |
| CR43358 | -0.62 | 0.00029274 |
| CG31233 | -0.62 | 1.06E-05 |
| Elo68alpha | -0.63 | 1.11E-05 |
| Jon74E | -0.64 | 2.52E-06 |
| TotC | -0.65 | 2.90E-05 |
| CG6295 | -0.65 | 1.26E-06 |
| CG5773 | -0.66 | 9.58E-06 |
| TotA | -0.69 | 2.00E-07 |
| CG7203 | -0.71 | 6.93E-05 |
| CG15155 | -0.72 | 8.99E-08 |
| Lsp1beta | -0.73 | 1.35E-05 |
| CG13026 | -0.80 | 0.002376329 |
| CG12057 | -0.80 | 1.14E-07 |
| CG11459 | -0.80 | 1.75E-05 |
| CG7214 | -0.84 | 0.000120478 |
| Diedel3 | -0.86 | 3.34E-11 |
| CG1946 | -0.89 | 6.90E-07 |
| CG34327 | -0.91 | 0.000296155 |
| Jon25Bii | -1.02 | 9.15E-14 |
| CG8147 | -1.07 | 8.99E-08 |
| Jon25Bi | -1.19 | <2.2E-14 |
| CG17192 | -1.30 | <2.2E-14 |
| mt:tRNA:V | -1.41 | 0.007674448 |

* Reads aligning to regions coding >1 gene are listed using the FlyBase IDs for the multiple genes.

** Fold-changes calculated by DESeq from expression coefficients.

*** Benjamini-Hochberg correction, P-value for microbiota accounting for sequencing lane and *Drosophila* genotype.
