# Supplementary material for: The Drosophila transcriptional network is structured by microbiota
Source: BMC Genomics. 2016 Nov 25;17:975. doi: 10.1186/s12864-016-3307-9 (PMC5124311; doi:10.1186/s12864-016-3307-9)
Supplement: Additional file 6: Table S4. — Frequency of data permutations with dispersion statistic greater than observed, within individual modules and between pairs of modules. Permutation tests suggesting significant changes (≤50/1000 permutations﻿ i.e., p ≤0.05) are underscored. (DOCX 15 kb) [file 12864_2016_3307_MOESM6_ESM.docx]

| Table S4. Frequency of data permutations with dispersion statistic greater than observed, within individual modules and between pairs of modules. Permutation tests suggesting significant changes (≤50/1000 permutations) are underscored. | | | | | | | | | | | |
| --- | --- | --- | --- | --- | --- | --- | --- | --- | --- | --- | --- |
| Module number | 1 / turquoise | 2 / blue | 3 / brown | 4 / yellow | 5 / green | 6 / red | 7 / black | 8 / pink | 9 / magenta | 10 / purple | 11 / greenyellow |
| 1 / turquoise | 514 | 541 | 151 | 1000 | 1000 | 1000 | 1000 | 714 | 373 | 784 | 1000 |
| 2 / blue |  | 1000 | 987 | 1000 | 1000 | 1000 | 525 | 831 | 1000 | 381 | 720 |
| 3 / brown |  |  | 1000 | 1000 | 1000 | 1000 | 139 | 88 | 1000 | 675 | 991 |
| 4 / yellow |  |  |  | **11** | 1000 | 198 | 928 | 187 | 848 | 1000 | 1000 |
| 5 / green |  |  |  |  | **0** | 730 | 962 | 336 | 1000 | 996 | **0** |
| 6 / red |  |  |  |  |  | **1** | 922 | 941 | 999 | 114 | 262 |
| 7 / black |  |  |  |  |  |  | 77 | 907 | **21** | 996 | 765 |
| 8 / pink |  |  |  |  |  |  |  | **0** | 440 | 1000 | 812 |
| 9 / magenta |  |  |  |  |  |  |  |  | 989 | 413 | 432 |
| 10 / purple |  |  |  |  |  |  |  |  |  | 85 | 998 |
| 11 / greenyellow |  |  |  |  |  |  |  |  |  |  | 408 |
