# Supplementary figures and images for: The Drosophila transcriptional network is structured by microbiota
Source: BMC Genomics. 2016 Nov 25;17:975. doi: 10.1186/s12864-016-3307-9 (PMC5124311; doi:10.1186/s12864-016-3307-9)

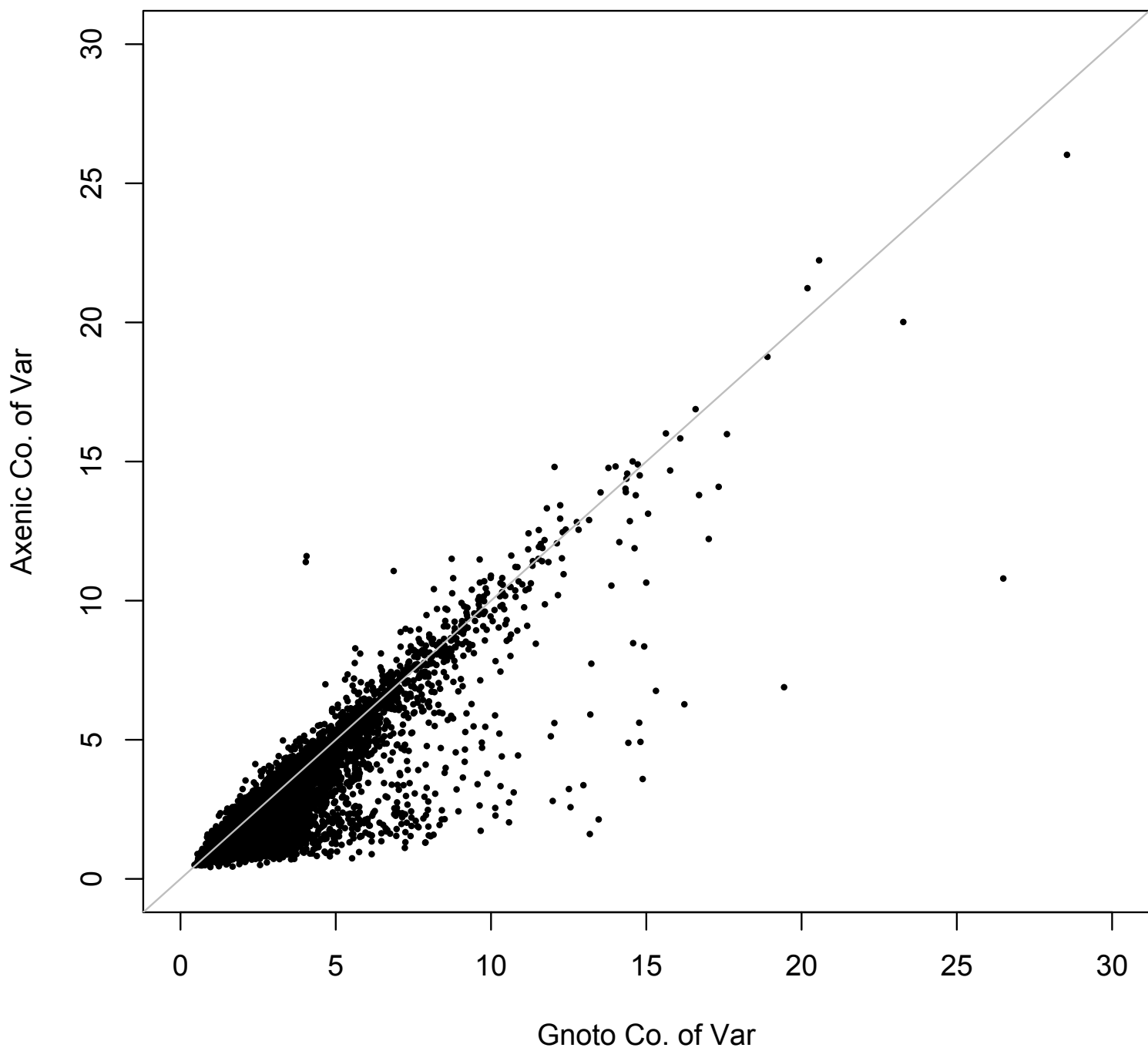

Supplement: Additional file 7: Figure S2. — Coefficients of variation per gene in axenic and gnotobiotic flies. Coefficients of variation across all lines were calculated per gene. The grey line indicates the null of equivalence between the two conditions. (PDF 875 kb) [file 12864_2016_3307_MOESM7_ESM.pdf]

Mean variance-stabilized expression

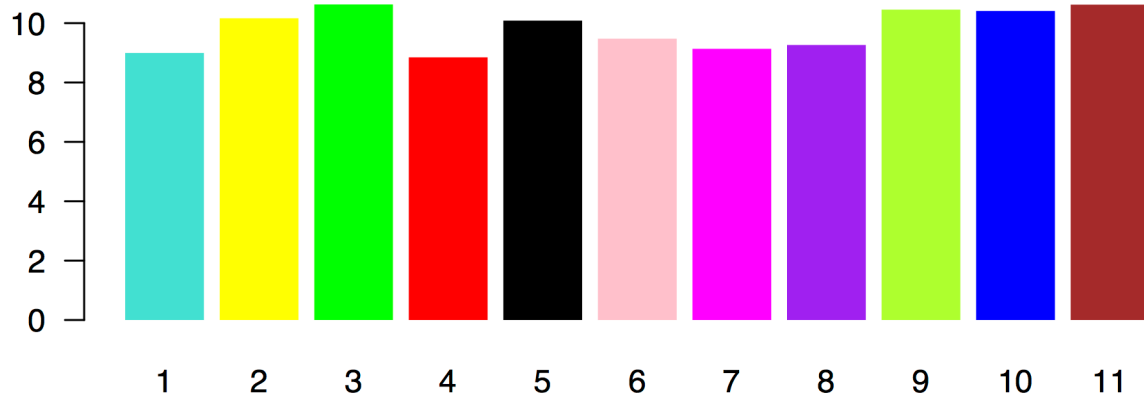

Mean variance-stabilized expression

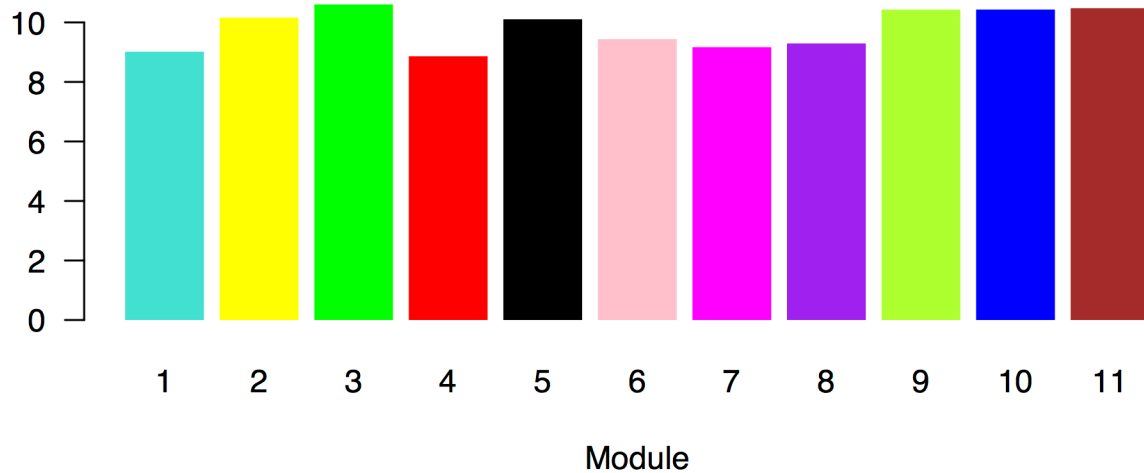

Supplement: Additional file 8: Figure S3. — Mean expression per transcriptional module in axenic and gnotobiotic conditions. Each bar shows mean expression of all genes assigned to each given transcriptional module. Color coding corresponds to Fig. 1b. (PDF 177 kb) [file 12864_2016_3307_MOESM8_ESM.pdf]
