# Supplementary material for: The Drosophila transcriptional network is structured by microbiota
Source: BMC Genomics. 2016 Nov 25;17:975. doi: 10.1186/s12864-016-3307-9 (PMC5124311; doi:10.1186/s12864-016-3307-9)
Supplement: Additional file 10: Table S6. — Drosophila lines used in this study. (DOCX 13 kb) [file 12864_2016_3307_MOESM10_ESM.docx]

Table S6 *Drosophila* strains used in this study

| Line name | Collected from |  |
| --- | --- | --- |
| (a)_Outbred Laboratory Stock | | |
| CantonS | USA |  |
| (b) Drosophila Genetic Reference Panel | | |
| RAL_306 | North Carolina, USA |  |
| RAL_318 |  |  |
| RAL_42 |  |  |
| RAL_555 |  |  |
| (c) Global Diversity Lines | | |
| N01 | Netherlands |  |
| N04 |  |  |
| N15 |  |  |
| ZW184 | Zimbabwe |  |
| I16 | New York, USA |  |
| I34 |  |  |
| I38 |  |  |
| B04 | Beijing, China |  |
| B10 |  |  |
| B17 |  |  |
| T01 | Tasmania, Australia |  |
| T05 |  |  |
| T29A |  |  |

Lines provided by (a) M. Wolfner, (b) B. Lazzaro, and (c) A. Clark
