# Supplementary material for: The Drosophila transcriptional network is structured by microbiota
Source: BMC Genomics. 2016 Nov 25;17:975. doi: 10.1186/s12864-016-3307-9 (PMC5124311; doi:10.1186/s12864-016-3307-9)
Supplement: Additional file 11: Table S7. — Adapter sequences used for RNA sequencing. (DOCX 12 kb) [file 12864_2016_3307_MOESM11_ESM.docx]

Table S7. TruSeq RNA Sample Prep Kit v2 Set A Indexed Adapter Sequences

| Adapter | Sequence |
| --- | --- |
| AR002 | CGATGT(A) |
| AR004 | TGACCA(A) |
| AR005 | ACAGTG(A) |
| AR006 | GCCAAT(A) |
| AR007 | CAGATC(A) |
| AR012 | CTTGTA(A) |
| AR013 | AGTCAA(C) |
| AR014 | AGTTCC(G) |
| AR015 | ATGTCA(G) |
| AR016 | CCGTCC(C) |
| AR018 | GTCCGC(A) |
| AR019 | GTGAAA(C) |
