# Supplementary material for: The Drosophila transcriptional network is structured by microbiota
Source: BMC Genomics. 2016 Nov 25;17:975. doi: 10.1186/s12864-016-3307-9 (PMC5124311; doi:10.1186/s12864-016-3307-9)
Supplement: Additional file 3: Figure S1. — Tissue tropism of differential gene expression in axenic and gnotobiotic flies. For the set of 177 genes that were differentially expressed between axenic and gnotobiotic flies, the bars show the frequency of genes which were enriched in each given tissue relative to the whole body in the FlyAtlas dataset. (PDF 103 kb) [file 12864_2016_3307_MOESM3_ESM.pdf]

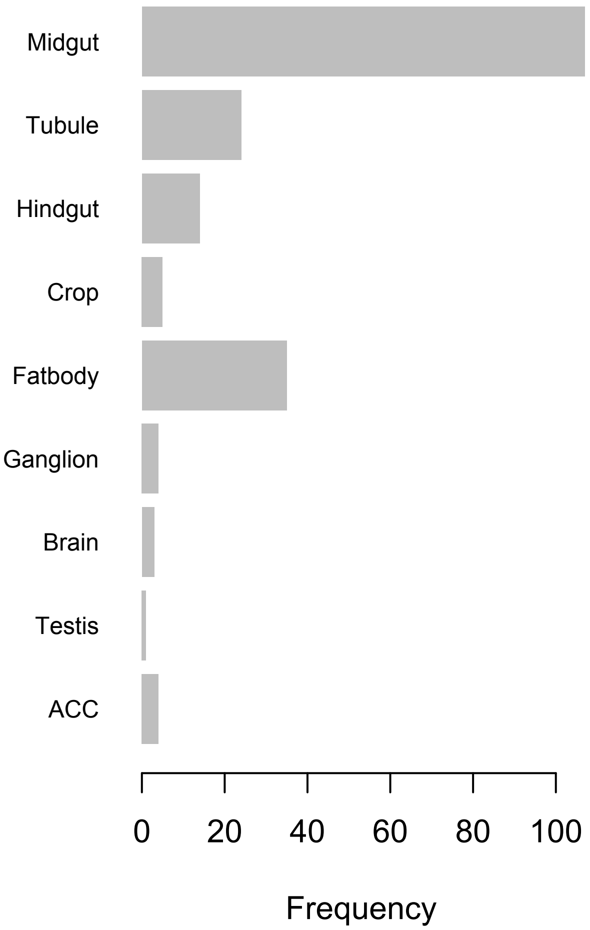
Midgut
Tubule
Hindgut
Crop
Fatbody
Ganglion
Brain
Testis
ACC
0
20
40
60
80
100
Frequency
